# Establishment of a N1-methyladenosine-related risk signature for breast carcinoma by bioinformatics analysis and experimental validation

**DOI:** 10.1007/s12282-023-01458-1

**Published:** 2023-05-13

**Authors:** Leilei Li, Wenhui Yang, Daqi Jia, Shiqi Zheng, Yuzhe Gao, Guanghui Wang

**Affiliations:** 1 Department of Pathology, Kunming Medical University, Kunming, Yunnan 650500 People’s Republic of China; 2 Department of Digestive Oncology, Cancer Center, Shanxi Bethune Hospital, Shanxi Academy of Medical Sciences, Tongji Shanxi Hospital, Third Hospital of Shanxi Medical University, Taiyuan, Shanxi 030032 People’s Republic of China; 3 Department of Breast Surgery, Guizhou Provincial People’s Hospital, Guiyang, Guizhou 550002 People’s Republic of China

**Keywords:** Breast invasive carcinoma, m^1^A-related regulators, Prognosis, Risk model, Immune infiltration

## Abstract

**Objectives:**

Breast carcinoma (BRCA) has resulted in a huge health burden globally. N1-methyladenosine (m^1^A) RNA methylation has been proven to play key roles in tumorigenesis. Nevertheless, the function of m^1^A RNA methylation-related genes in BRCA is indistinct.

**Methods:**

The RNA sequencing (RNA-seq), copy-number variation (CNV), single-nucleotide variant (SNV), and clinical data of BRCA were acquired via The Cancer Genome Atlas (TCGA) database. In addition, the GSE20685 dataset, the external validation set, was acquired from the Gene Expression Omnibus (GEO) database. 10 m^1^A RNA methylation regulators were obtained from the previous literature, and further analyzed through differential expression analysis by rank-sum test, mutation by SNV data, and mutual correlation by Pearson Correlation Analysis. Furthermore, the differentially expressed m^1^A-related genes were selected through overlapping m^1^A-related module genes obtained by weighted gene co-expression network analysis (WGCNA), differentially expressed genes (DEGs) in BRCA and DEGs between high- and low- m^1^A score subgroups. The m^1^A-related model genes in the risk signature were derived by univariate Cox and least absolute shrinkage and selection operator (LASSO) regression analyses. In addition, a nomogram was built through univariate and multivariate Cox analyses. After that, the immune infiltration between the high- and low-risk groups was investigated through ESTIMATE and CIBERSORT. Finally, the expression trends of model genes in clinical BRCA samples were further confirmed by quantitative real-time PCR (RT‒qPCR).

**Results:**

Eighty-five differentially expressed m^1^A-related genes were obtained. Among them, six genes were selected as prognostic biomarkers to build the risk model. The validation results of the risk model showed that its prediction was reliable. In addition, Cox independent prognosis analysis revealed that age, risk score, and stage were independent prognostic factors for BRCA. Moreover, 13 types of immune cells were different between the high- and low-risk groups and the immune checkpoint molecules TIGIT, IDO1, LAG3, ICOS, PDCD1LG2, PDCD1, CD27, and CD274 were significantly different between the two risk groups. Ultimately, RT-qPCR results confirmed that the model genes MEOX1, COL17A1, FREM1, TNN, and SLIT3 were significantly up-regulated in BRCA tissues versus normal tissues.

**Conclusions:**

An m^1^A RNA methylation regulator-related prognostic model was constructed, and a nomogram based on the prognostic model was constructed to provide a theoretical reference for individual counseling and clinical preventive intervention in BRCA.

**Supplementary Information:**

The online version contains supplementary material available at 10.1007/s12282-023-01458-1.

## Introduction

Breast carcinoma (BRCA) is the most common malignant tumor in women [1]. The percentage of women with BRCA is steadily increasing, with an estimated 11.7% of all cancer cases or 2.3 million new cases in 2020 [1, 2]. BRCA can be mainly classified into luminal A, luminal B, HER-2 positive, and triple-negative breast cancer (TNBC) according to the expression of ER, PR, HER-2, and Ki-67. The different subtypes may lead to different responses to clinical treatment and different prognoses [3]. Great improvements in the early diagnosis and comprehensive treatment of breast cancer have been made in recent years, but breast cancer is still the leading cause of cancer death in women [2]. Therefore, there is an urgent need to develop new therapeutic and prognostic targets for BRCA.

Growing evidence has revealed that RNA chemical modifications have important functions in vital cellular processes, such as cell differentiation, circadian rhythm maintenance, cell signaling, and protein production [4–6]. RNA methylation is one of the most common RNA chemical modification patterns observed during posttranscriptional RNA epigenetic modifications, including N3-methylcytosine (m3C), N1-methyladenosine (m^1^A), N6-methyladenosine (m6A), and 5-methylcytosine (m5C) [7–11]. The N1 position where the methyl group is attached to adenosine is m^1^A [12] and is prevalent in rRNA, mRNA, tRNA, and mitochondrial transcripts. Most m^1^A is found in GC-rich sequences with a highly structured 5-untranslated region (UTR) near the mRNA translation start site [8]. Studies have shown that m^1^A dysregulation affects RNA structural stability, protein interactions, folding, cell proliferation, and cell death [8, 13]. M^1^A methylation regulators are composed of “eliminate decoders” (ALKBH3, ALKBH1), “encoders” (TRMT61B, TRMT10C, TRMT6, TRMT61A), and “code readers” (YTHDC1, YTHDF1, YTHDF2, YTHDF3) [14–17]. It has been demonstrated that dynamic regulation of m^1^A in response to physiological stress and abnormal expression of m^1^A regulators are associated with tumorigenesis and cancer recurrence [18]. Studies have illustrated that changes in m^1^A-related genes are closely related to the progression of bladder cancer [19]. The expression of ALKBH1 is closely related to the poor prognosis of lung adenocarcinoma (LUAD) [20]. A study by Couch et al. demonstrated that TRMT61B is closely associated with ER-negative breast cancer [21].

However, the impact of m^1^A-related genes on breast cancer prognosis remains unclear. Therefore, this study mainly used bioinformatics analysis to investigate the effect of m^1^A-related genes on the prognosis of breast cancer patients. A total of 6 m^1^A-related genes (MEOX1, COL17A1, FREM1, CD1C, TNN, and SLIT3) were selected as prognostic m^1^A-related biomarkers to build the risk model. The validation results of the risk model showed that its prediction was reliable. Finally, the experimental results demonstrate the reliability of the bioinformatics analysis. This research may provide new ideas for the diagnosis and treatment of BRCA.

## Materials and methods

### Data source

The transcriptome (1104 BRCA samples and 114 normal samples), copy-number variations (CNVs), single-nucleotide variants (SNVs), and clinical data of BRCA were downloaded from the TCGA database (https://portal.gdc.cancer.gov/). After removing the samples without survival status and invalid survival time, a total of 1069 BRCA samples with complete survival information were obtained for subsequent risk model construction. Moreover, the GSE20685 dataset, the external validation set, was retrieved from the GEO database (https://www.ncbi.nlm.nih.gov/geo), containing the expression profile data of 115 BRCA samples with survival information. Finally, 10 m^1^A RNA methylation regulators were obtained from the literature of Wu et al. [22], including ALKBH1, ALKBH3, TRMT10C (AKA: RG9MTD1), YTHDF2, TRMT61A, TRMT61B, YTHDC1, TRMT6, YTHDF1, and YTHDF3.

### CNV and functional enrichment analyses of m^1^A RNA methylation regulators

Considering the important role of various CNV regions in lymph-node metastasis in triple-negative breast cancer patients [23], the genetic variation of N6-methyladenosine (m6A)-related regulators, that is, CNV data, expression differences, and mutations, has been investigated in BRCA [24]. In the current study, similarly, the R package was employed for the 10 m^1^A-related regulators to initially seek their CNV types and frequencies and chromosomal assignments. In addition, based on transcriptome data, the expression patterns of 10 m^1^A-related regulators were compared between BRCA samples and controls using the rank-sum test and visualized by ggplot2 (Version 3.3.3). For the exploration of the biological function and signaling pathway of these m^1^A-related regulators, Gene Ontology (GO) enrichment analysis was further utilized for the 10 m^1^A-related regulators by ClusterProfiler (version 3.18.0) [25] in the R package with adj.*p* < 0.05 and count ≥ 1 as the selection standard, and the results were visualized in a bar chart and bubble diagram by enrichplot (version 1.10.2) [26].

### Survival analysis and immune infiltration of 10 m^1^A-related regulators

The m^1^A score of each of 1069 BRCA samples was calculated by single sample Gene Set Enrichment Analysis (ssGSEA) of Gene Set Variation Analysis (GSVA) [27, 28]. Then, the survival time and survival data were extracted, and the survminer package (version 0.4.9) was utilized to calculate the optimal threshold of m^1^A scores to separate BRCA samples into m^1^A score high-risk and low-risk subgroups according to previous methods [29]. The survival package (Version 3.2–11) [30] was used for the survival analysis of the two subgroups, a survival curve was drawn, and *p* < 0.05 was considered statistically significant.

In addition, the immune infiltration was analyzed in the two score subgroups [31]. First, the ESTIMATE algorithm was applied for the stromal scores, immune scores, and ESTIMATE composite scores of each sample in two score subgroups. The comparison of the three scores between the two m^1^A score subgroups was conducted through the rank-sum test and visualized in violin plots. Moreover, CIBERSORT was further performed on each sample in both subgroups to compute the proportions of 22 types of immune cells, and the correlations of the immune cells were computed by Pearson correlation analysis [32].

### Screening of m^1^A-related Genes

In contrast to the previous studies of BRCA, disease status (BRCA/normal) was used as the trait data of weighted gene co-expression network analysis (WGCNA) (Version 1.70-3), which was performed to filter the most relevant m^1^A modules and genes in this study [33]. Initially, the expression values of genes were computed, and genes with expression values greater than 1 were selected for cluster analysis. The cluster analysis of samples aimed to determine whether outlier samples needed to be removed. Next, the adjacency and similarity between genes were calculated. Then, the dynamic tree cutting algorithm was performed to divide modules. Based on the following settings, the minimum number of genes per module was 200, MEDissThres = 0.2. Finally, genes in the key modules that met the requirements of 丨GS丨 > 0.2, and 丨MM丨 > 0.6 were regarded as m^1^A-related genes.

### Differentially expressed m1A-related genes

The limma package (Version 3.44.3) [34] was applied with the criteria of adj.*p* < 0.05 and |Log2FC|≥ 1 to screen out the differentially expressed genes (DEGs) between 1104 BRCA samples and 114 normal samples. Similarly, DEGs between m^1^A score high-risk and low-risk subgroups were identified as well, which exhibited the differences in gene expression between BRCA cohorts with different prognoses according to the m^1^A score. In addition, the VennDiagram package (Version 1.6.20) was utilized to overlap DEGs in BRCA samples, DEGs in m^1^A score high-risk and low-risk subgroups, and the m^1^A-related module genes obtained by WGCNA (the downregulated DEGs in BRCA were intersected with the downregulated genes in the m^1^A score high-risk subgroup and key module genes, and vice versa) to acquire the differentially expressed m^1^A-related genes.

### Construction and validation of the m^1^A-related prognosis model

First, based on the ratio of 7:3, 749 and 320 samples of the 1069 BRCA samples were treated as the training set and internal validation set, respectively. Moreover, after extracting the expression data of differentially expressed m^1^A-related genes from the training set, the overall survival (OS) and other clinical information were combined with the extracted expression data to further obtain clinical expression data of BRCA samples. Then, the risk model was built by univariate Cox analysis and least absolute shrinkage and selection operator (LASSO) logistic regression [35]. To be more specific, univariate Cox analysis was applied to screen out the differentially expressed m^1^A-related genes with *p* < 0.05 by the survival package (version 3.2-11). LASSO logistic regression analysis was performed with the setting of family to Cox, to the screened differentially expressed m^1^A-related genes for constructing the risk model. At the meantime, Kaplan–Meier (K–M) survival analysis was conducted to evaluate the difference in OS of TCGA-BRCA cohorts with different expression patterns of these model genes, as described by Zhang et al. [36].

Moreover, the RiskScore of every BRCA patient was computed by the risk coefficient (${coef}_{i}$) of the model genes obtained by LASSO and their respective expression levels (${x}_{i}$), with the formula RiskScore = $\sum\nolimits_{n = 1}^{n} {coef_{i} } * x_{i}$ [37]. After separating BRCA patients into high- or low-risk (score) groups based on the median RiskScores, the overall survival curves were plotted for the groups by survminer (version 0.4.8). The risk model efficacy was further assessed by the area under the curve (AUC) of the receiver-operating characteristic (ROC) curves37. The 1-, 3- and 5-year survival time node ROC curves were plotted by the survival ROC package (Version 3.1-12) for the risk model. Moreover, the same evaluation procedures were employed for both the internal and external validation (GSE20685) sets to further determine the effectiveness of the risk model.

### Establishment of a nomogram

To detect the prognosis of the risk model and clinical factors, correlation analysis was performed between clinicopathological characteristics (age, T, N, M, sex, subtype, vital, and stage) of the 1069 BRCA samples and the RiskScore. The differences in subgroups of clinicopathological characteristics [age (> 60 and ≤ 60), T (T1, T2, T3, T4), M (M0, M1), N (N0, N1, N2, N3), sex (male, female), subtype (BRCA_LumA, BRCA_LumB, BRCA_Normal, BRCA_Basal, BRCA_Her2), and Vital (Alive, Dead)] between the low- and high-risk groups were examined by the Chi-square test. In addition, the rank-sum test was further utilized to compare differences in RiskScores expression among the different subgroups of clinicopathological characteristics [38]. Considering the vital significance of the intrinsic subtype of BRCA in prognosis, risk stratification analysis was further conducted to investigate the utilization of the risk model in patients with different intrinsic subtypes of BRCA [39].

Next, univariate Cox independent prognostic analysis was further employed for the clinicopathological characteristics and RiskScore to investigate the prognosis of the clinicopathological characteristics and the risk model. Subsequently, clinicopathological characteristics with *p* < 0.05 were regarded as factors for multivariate Cox independent prognostic analysis.

Then, the rms function (version 6.2-0) in the R package was employed on the 1069 BRCA samples to construct the nomogram to further predict BRCA patient 1-, 3-, and 5-year survival probabilities according to the total score of independent factors screened by the Cox analyses, and the nomogram was verified by the overall calibration curve [40]. In addition, the effectiveness comparison between the risk model and nomogram was evaluated by decision curve analysis (DCA).

### Effects of the risk model on immune heterogeneity

The estimate package (version 1.0.13) was performed on the 1069 BRCA samples to detect the immune differences between the two risk groups. The immune infiltration of both stromal and immune cells in a tumor sample can be obtained by the ESTIMATE algorithm, which is presented as stromal scores, immune scores, and ESTIMATE composite scores [41]. Furthermore, the abundances of 22 types of immune cells were calculated in each BRCA sample by the CIBERSORT algorithm (Version 1.03) [42] with *p* < 0.05 as the selection standard. Then, the rank-sum test was used to compare the proportions of 22 immune cells between the high- and low-risk groups. In addition, the correlation between risk model genes and differential immune cells was detected through Pearson correlation.

In this study, the rank-sum test was employed to compare the differences between the two risk groups in both the expression data of 24 human leukocyte antigen (HLA) genes extracted from a previous publication by Yue et al. [43] and the expression data of nine immune checkpoint molecules (LAG3, ICOS, TIGIT, CD274, PDCD1, IDO1, CD27, PDCD1LG2, and HAVCR2).

### Analysis of risk model gene mutations

Eventually, the SNVs of each risk model gene in BRCA were analyzed according to the SNV data, and their mutation frequencies were presented as a waterfall diagram by maftools R package [44].

### Total RNA extraction and quantitative real-time PCR (qRT‒PCR) analysis

All clinical samples were obtained from the Department of Breast Surgery, Guizhou Provincial People's Hospital, and all patients signed informed consent. The clinical characteristics of the patients are shown in Table 1. Total RNA was extracted from BRCA tissues and healthy controls using RNA extraction kits (Promega, Shanghai, China) in accordance with the manufacturer's instructions. Then, cDNA synthesis was conducted on 2 μg of each sample using a fixed one-step RT-PCR kit (Promega, Shanghai, China). The SYBR Green Super Mix system was applied to perform qRT‒PCR. Gene expression was evaluated for three biological replicates, and the 2^−ΔΔCT^ method was employed to analyze the relative changes in gene expression. GAPDH was utilized as a control. The primer sequences used in this paper are listed in Table 2.

**Table 1:** Clinical features of patients

Features	Variables	No. (%)
Age	** < **35	8 (40)
	** ≥ **35	12 (60)
Gender	Female	20 (100)
Molecular subtype	Luminal A	5 (25)
	Luminal B	5 (25)
	HER-2(+)	5 (25)
	TNBC	5 (25)
Lymphatic metastasis	Yes	16 (80)
	No	4 (20)
Distant metastasis	Yes	2 (10)
	No	18 (90)

**Table 2:** The primer sequences for qPCR

Primers for validated Genes Gene	Prime sequence (5′–3′) Forward reverse	
GAPDH MEOX1	TATGACAACAGCCTCAAGAT CCAACTGGCACTTCCCTGTCTC	AGTCCTTCCACGATACCA TCTCCGCCTGGATGATTTCTTC
COL17A1	GCTCCAGTGGCAACTCTTCTC	CTCTCGTGTTTGACTCCGTCC
FREM1	GTGAATGGGAGAGTGTGGGAAG	GCAAGAGTGTGATACGAGGAGC
CD1C TNN SLIT3	TGAAGTACAGGTGAAAGCGG GAGATGTTCCGCTTCCCTATG ACTGTTTGATGGGCTGGTGTC	CATCCAGGAGACCCAAGAGA TGATGTTCTGTTCCTCCCTGG TGGGCTAAGTGGAGTGTCTGG

## Results

### CNV and expression of m^1^A RNA methylation regulators

**Fig. 1:**
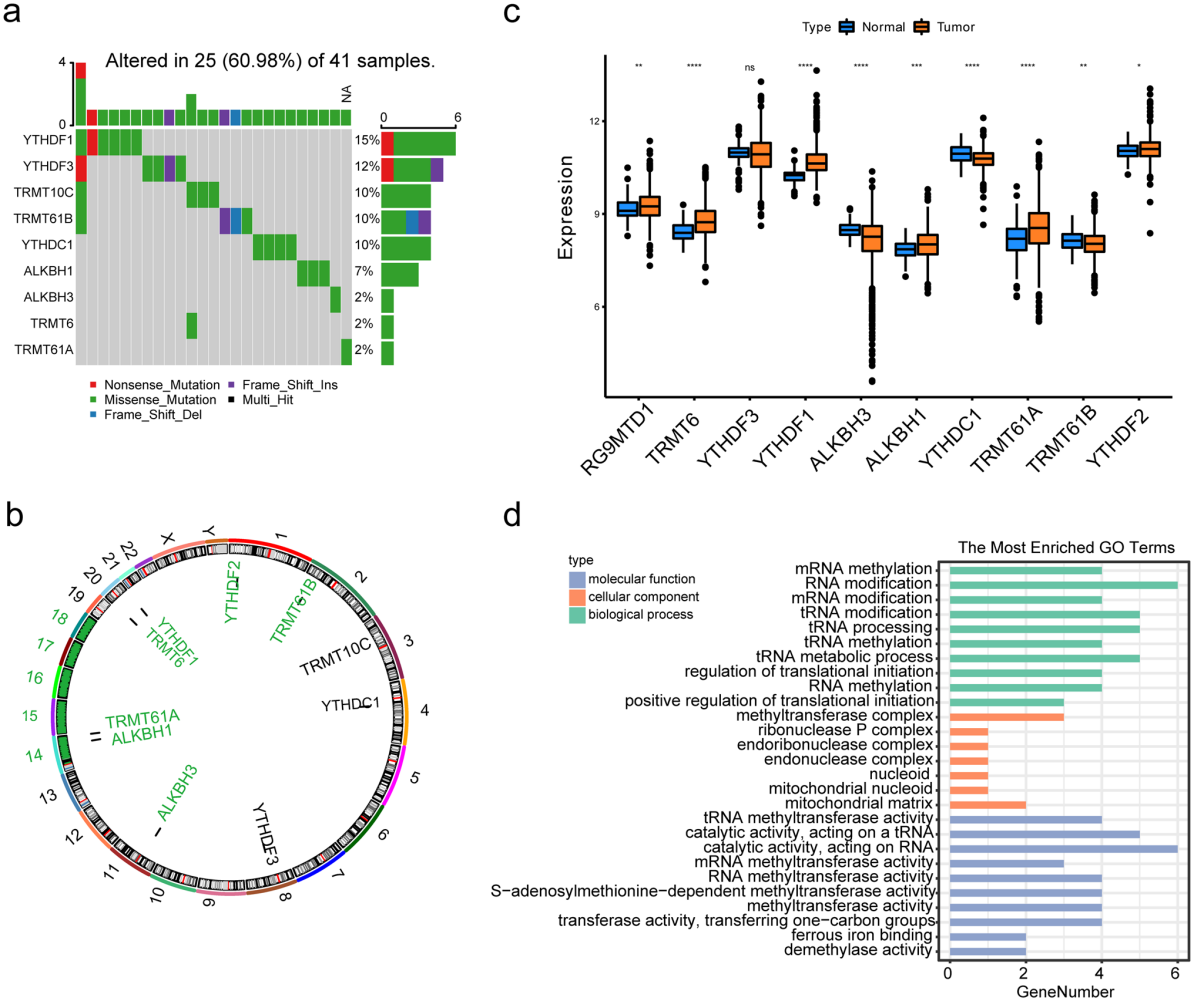
CNV and expression of m^1^A RNA methylation regulators. **a** Waterfall plot of CNV types and frequencies of 10 m^1^A RNA methylation regulators. **b** Location of CNV alteration of the 10 m^1^A regulators on chromosomes. **c** Expression of 10 m^1^A methylation regulators between breast tumor tissue and breast normal tissue (* represents *p* < 0.05, **represents *p* < 0.001, ***represents *p* < 0.001, and ****represents *p* < 0.0001). **d** Bar plot of enriched GO terms in 3 categories for the 10 m^1^A RNA methylation regulators

To explore the functional alteration and biological significance of m^1^A RNA methylation in BRCA development, the mutation rate of CNV and the expression levels of 10 m^1^A RNA methylation regulators were first evaluated. The waterfall diagram showed that 9 of 10 m^1^A RNA methylation regulators were mutated in the BRCA samples, and YTHDF1 was the gene with the highest mutation frequency of 15% (Fig. 1a). For the chromosomal assignment results, it was revealed that 10 m^1^A RNA methylation regulators were mainly located on chromosomes 1, 2, 3, 4, 8, 11, 14, and 22 (Fig. 1b). Besides, copy-number amplification occurred in 8 regulators except ALKBH1 and TRMT61A which experienced copy-number deletions (Table 3). However, in general, the probabilities of CNV among the 10 m^1^A RNA methylation regulators in BRCA samples were low.

**Table 3:** The CNV situation of m^1^A regulators

m^1^A	Amp	Del	Stable
YTHDF2	4/1081	0	1077/1081
TRMT61B	7/1081	0	1074/1081
TRMT10C	14/1081	0	1067/1081
YTHDC1	13/1081	0	1068/1081
YTHDF3	172/1081	0	909/1081
ALKBH3	14/1081	0	1067/1081
ALKBH1	5/1081	1/1081	1076/1081
TRMT61A	10/1081	2/1081	1071/1081
TRMT6	25/1081	0	1056/1081
YTHDF1	96/1081	0	985/1081

From the perspective of the gene expression patterns of these 10 m1A RNA methylation regulators in BRCA, the boxplot of the rank-sum test suggested that except for YTHDF3, the expression levels of the other nine m^1^A RNA methylation regulators showed significant differences between tumor and normal samples (Fig. 1c), indicating the potential correlation of m^1^A RNA methylation and BRCA.

Next, using the enrichment analysis targeted the m^1^A RNA methylation regulators, the GO enrichment results indicated that in the BP process, these regulators were mostly significantly correlated with the methylation and modification of RNA, tRNA, and mRNA such as positive regulation of translation initiation, RNA methylation, tRNA metabolism process, tRNA methylation, tRNA processing, tRNA modification, mRNA modification, and mRNA methylation. From the perspective of cellular components (CC), they were significantly correlated with the mitochondrial matrix, mitochondrial nucleoid, nucleus, endonuclease complex, endoribonuclease complex, ribonuclease P complex, and methyltransferase complex. Additionally, demethylase activity, methyltransferase activity, transferase activity, RNA methyltransferase activity, and S-adenosylmethionine-dependent methyltransferase activity were mainly enriched in the molecular functions (MF) (Fig. 1d).

### Prognostic value and immune correlation analysis of m^1^A RNA methylation regulators of BRCA

**Fig. 2:**
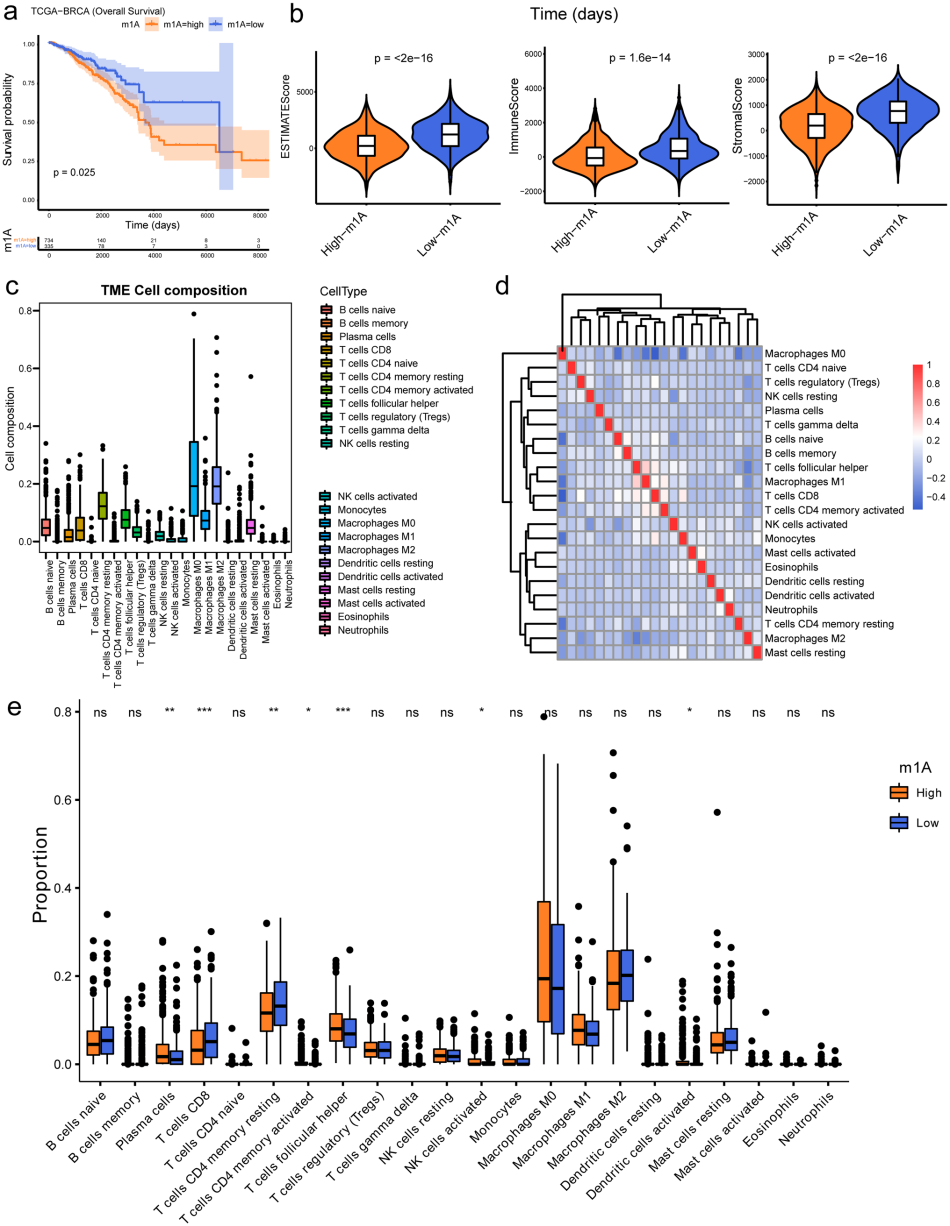
The grouping of m^1^A regulators and their immune infiltration relationship. **a** The K–M curves for BRCA patients in two m^1^A score subgroups. **b** Violin plots of the immune, stromal, and ESTIMATE score differences between the two m^1^A score subgroups. **c** Boxplot of TME cell composition. **d** Heatmap of the correlation between the 22 types of immune cells. **e** Boxplot of immune cell infiltration in the two m^1^A score subgroups

Following the m^1^A score of 1069 BRCA samples was calculated using ssGSEA, the m^1^A score high-risk and low-risk subgroups were generated according to the optimal threshold of m^1^A scores. Differences in OS and immune infiltration states between two subgroups were revealed using K–M survival analysis and the immune-related analyses. It was revealed that the prognosis of BRCA patients in the m^1^A score high-risk group was significantly worse (*p* = 0.025) (Fig. 2a). For the exploration of the immune infiltration, both ESTIMATE and CIBERSORT algorithms were employed. The ESTIMATE results illustrated that the m^1^A score low-risk group had significantly higher stromal scores, immune scores, and ESTIMATE composite scores (Fig. 2b). In addition, CIBERSORT results illustrated that M0 macrophages had a strong negative correlation with all the remaining immune cells. The proportions of seven types of immune cells (CD T cells, follicular helper T cells, activated NK cells, plasma cells, resting memory CD4 T cells, activated memory CD4 T cells, and activated dendritic cells) were significantly different between the two score subgroups (Fig. 2c–e).

### MElightgreen and MEblack with the strongest correlations to the m^1^A score

**Fig. 3:**
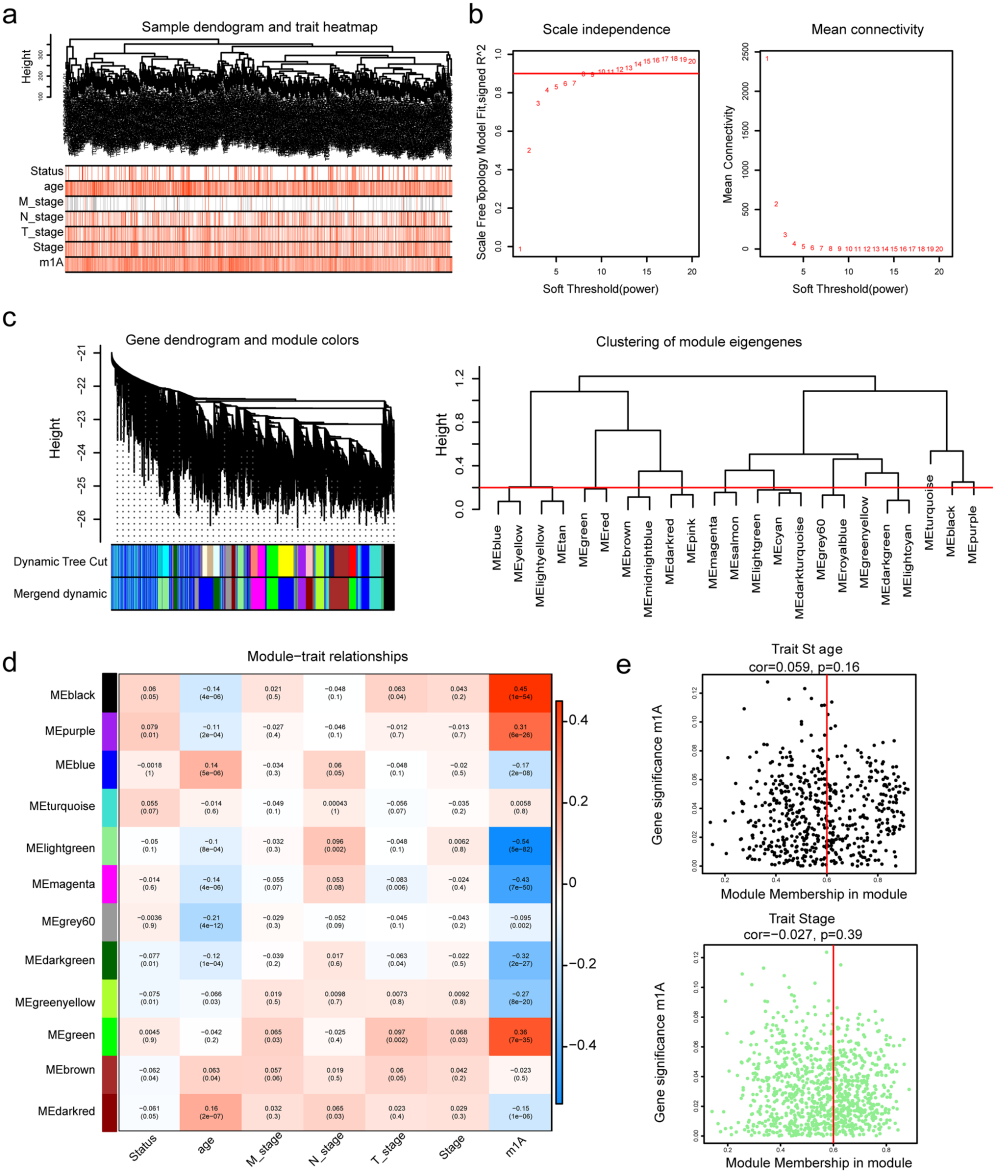
Screening of m^1^A-related genes. **a** The dendrogram and phenotypic trait heatmap of BRCA samples. **b** Soft threshold power analysis was implemented to obtain the scale-free fit index of the network topology, with a soft threshold power β of 8 hierarchical cluster analysis detection of the co-expression clusters was determined by WGCNA. **c** The upper part is the hierarchical clustering dendrogram of genes, and the lower part is the gene module. The genes clustered in the same branch are divided into the same module, and different colors represent different modules. **d** Heatmap of the correlation between different modules and m^1^A traits. The darker the color is, the higher the correlation. Red indicates a positive correlation and blue indicates a negative correlation. The number in the cell indicates the correlation and significance. The upper row is the correlation, the lower row is the p value, the left side is the module gene of different colors, and the color bar on the right side represents the correlation range. **e** Left: the correlation coefficient between the MEblack gene and modular traits was 0.52 (*p* < 0.05). Right: the correlation coefficient between the MElightgreen gene and the modular trait was 0.8 (*p* < 0.05)

Next, the genes associated with the m^1^A score were identified using WGCNA methods. The clustering analysis showed that there was no outlier sample, and the sorted staging data of m^1^A, T, N, M, age, and stage selected were used to construct a sample dendrogram and trait heatmap (Fig. 3a). Moreover, the optimal soft threshold analysis revealed that β was selected as 8 (Fig. 3b). On account of the optimal soft threshold, when 200 was set to the minimum number of genes in each gene module, 23 modules were acquired (Fig. 3c). Moreover, with MEDissThres set to 0.2 to merge similar modules analyzed by the dynamic tree cutting algorithm, 12 modules were obtained (Fig. 3c). Using m1A characteristics as well as other clinical characteristics as the clinical phenotypes of WGCNA, the correlation heatmap between modules and clinical phenotypes illustrated that of the 12 modules, MElightgreen (Cor = − 0.54, *p* = 5E−82) had the strongest negative correlation with m^1^A characteristics, while MEblack (Cor = 0.45, *p* = 1E–54) correlated the most negatively with m^1^A traits. Therefore, MElightgreen and MEblack were considered key modules related to m^1^A (Fig. 3d). As for, the correlations between m^1^A traits and genes in these key modules, it was showed that the correlation coefficient between MEblack genes and m^1^A traits was 0.52 (*p* < 0.05), and 220 of 574 genes in the MEblack module met the selection requirements. Furthermore, the correlation coefficient between the MElightgreen gene and m^1^A traits was 0.8, and 434 of 1000 genes in the MElightgreen were selected as m^1^A-related genes (Fig. 3e).

### Identification of differentially expressed m^1^A-related genes

**Fig. 4:**
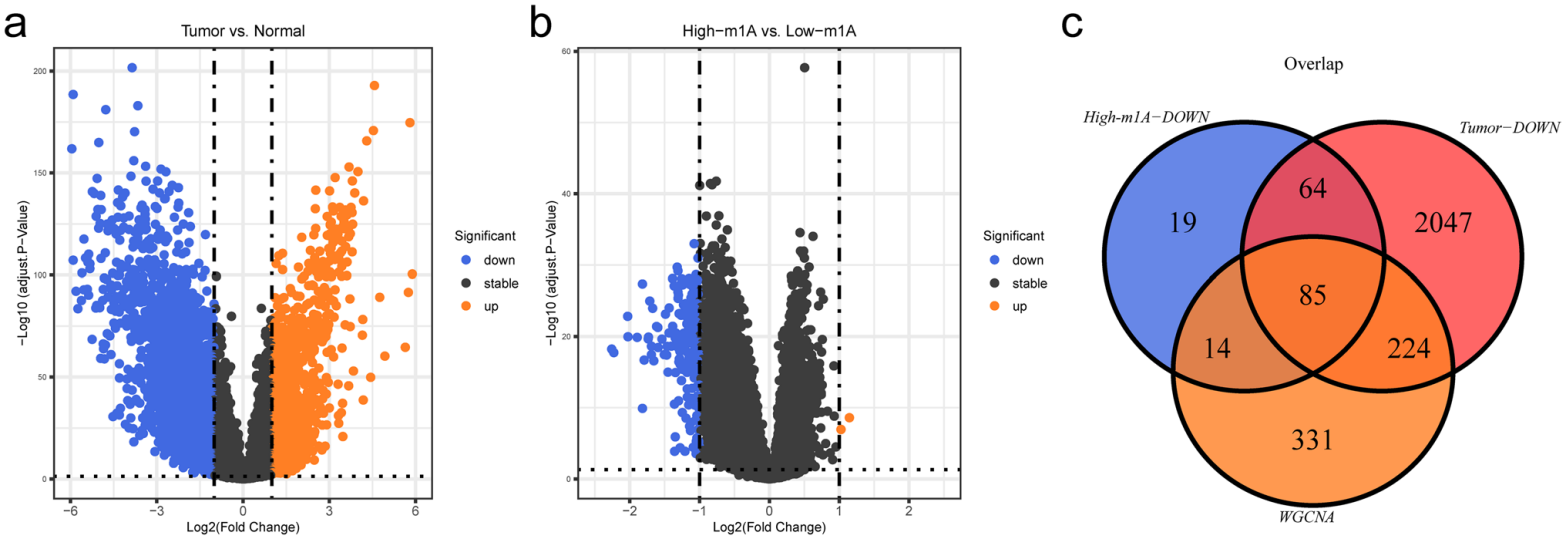
Identification of differentially expressed m^1^A-related genes. **a** Volcano plot of differentially expressed genes between 1104 BRCA samples and 114 normal samples The abscissa represents log_2_FC and the ordinate represents –log_10_ (ajust. *p*.value). Each dot in the figure represents a gene, the red dots represent up-regulated differentially expressed genes, the blue dots represent downregulated differentially expressed genes, and the black dots represent no significant differences in these genes. The transverse reference line represents –log_10_ (*p*.value = 0.05), and the longitudinal reference line represents log_2_FC =  ± 1. **b** Volcano plot of differentially expressed genes between the high-m^1^A and low-m^1^A score subgroups. **c** Venn diagram of downregulated genes in the high-m^1^A subgroup, downregulated DEGs in TumorBRCA samples, and m^1^A-related genes from WGCNA

To further explore the differences in gene expression between different subgroups, the differentially expressed analysis was conducted with adj.*p* < 0.05 and |Log_2_FC|≥ 1. A total of 3600 DEGs, of which 1180 were up-regulated and 2420 were downregulated genes in BRCA samples compared to controls, were collected **(**Fig. 4a). Similarly, 184 differentially expressed genes related to BRCA prognosis were found between the two m^1^A score risk subgroups, including 182 downregulated and 2 up-regulated genes in m^1^A score high-risk samples (Fig. 4b). Finally, the overlap analysis among DEGs in BRCA, DEGs in the high-m^1^A/low-m^1^A risk subgroups, and m^1^A-related genes showed that 85 differentially expressed m^1^A-related genes were selected (overlapping genes were only obtained in downregulated genes) (Fig. 4c).

### High efficiency of risk model based on 6 model genes

**Fig. 5:**
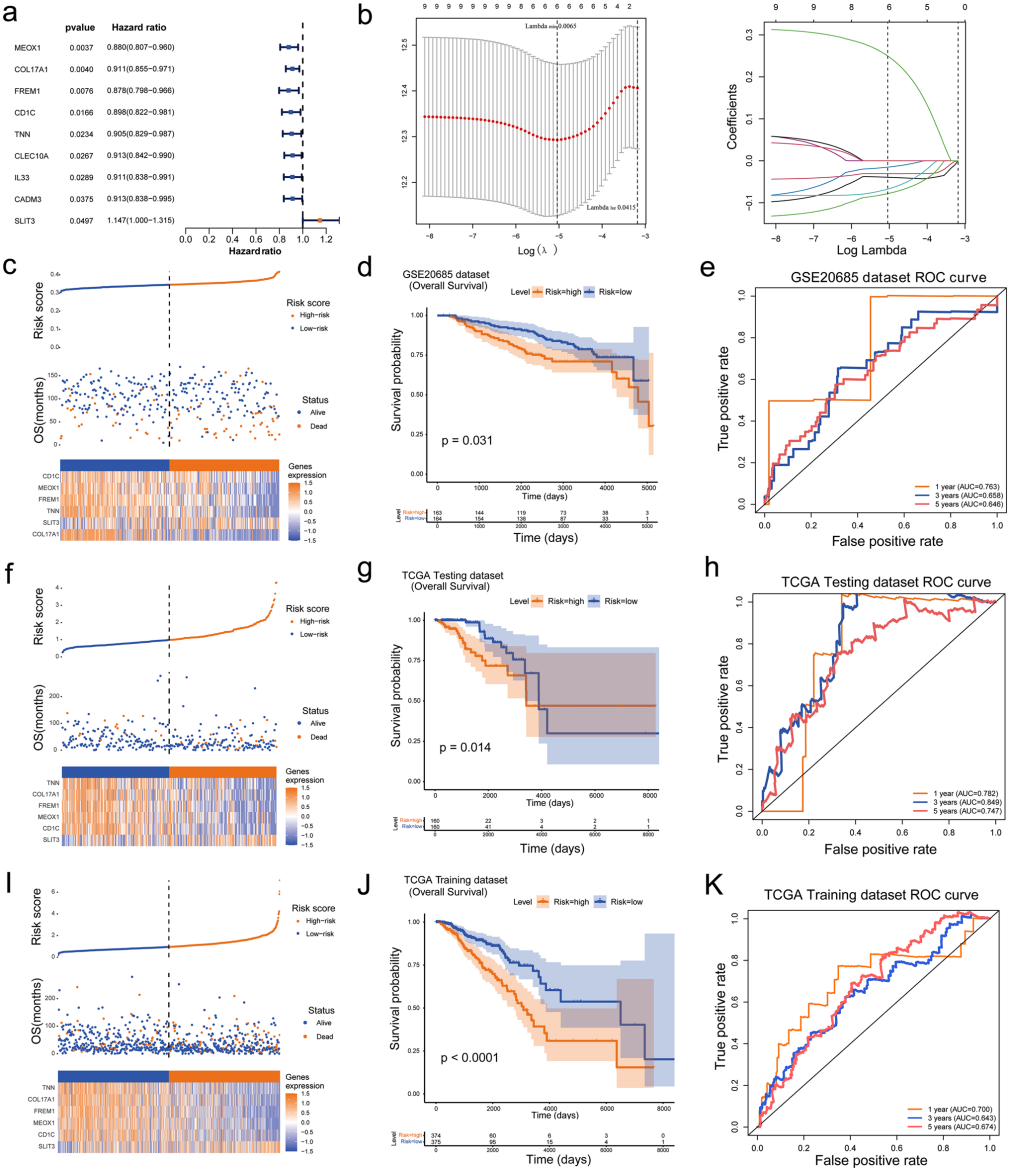
Construction and validation of the risk model. **a** Forest plot of univariate Cox regression analysis. **b** LASSO regression for the selection of characteristic parameters. Left: penalty graph of 6 characteristic variable coefficients. Right: in the LASSO logistic regression model, the best penalty coefficient lambda was selected using a fivefold cross-validation and minimization criterion. **c**, **f** and **i**: The risk curve and heatmap of 6 model genes for the training set, internal validation set and the external validation set, respectively. **d**, **g** and **j**: Survival analysis between patients in the high-risk group and the low-risk group for the training set, internal validation set, and the external validation set, respectively. **e**, **h**, and **k** ROC curves of 1-, 3-, and 5- year survival time nodes for the training set, internal validation set, and the external validation set, respectively

The expression data of 85 differentially expressed m^1^A-related genes were extracted from the training set to explore their prognostic value in BRCA. After combining these data with OS clinical information, forest map was drawn to visualize the univariate Cox analysis results, and nine differentially expressed m^1^A-related genes relevant to survival were identified including MEOX1, COL17A1, FREM1, CD1C, TNN, CLEC10A, IL33, CADM3, and SLIT3. Besides, SLIT3 was a risk factor (HR > 1) and the rest of the factors were protective factors (HR < 1) (Fig. 5a). The LASSO regression analysis results of these nine genes involved further suggested that 6 genes (MEOX1, COL17A1, FREM1, CD1C, TNN, and SLIT3) were screened out as model genes when the cross-validation error was lowest (lambda. min = 0.00397) (Fig. 5b). About the survival differences of these genes with distinct expressed patterns, the K–M analysis results preliminarily suggested that cohorts with low expression of COL17A1, FREM1, CD1C, and TNN had worse prognoses (Supplementary Fig. 1). Next, the RiskScore of the six model genes was estimated with the formula: RiskScore = 0.31 × SLIT3 + (− 0.04) × MEOX1 + (− 0.03) × COL17A1 + (− 0.1) × FREM1 + (− 0.03) × CD1C + (− 0.09) × TNN. The risk curve based on the RiskScore of 749 samples in training set showed that the high-risk patients experienced worse survival (Fig. 5c). The K–M curve demonstrated that the low-risk patients had a higher survival probability (Fig. 5d). Furthermore, the AUCs of the ROC curve in the training set were all greater than 0.6 at 1, 3, and 5 years, suggesting that the efficiency of the risk model was good (Fig. 5e). Moreover, the validation results of both the internal (test set) and external validation (GSE20685) sets all showed consistent results with those of the training set (Fig. 5f–k).

### Clinical correlation analysis

**Fig. 6:**
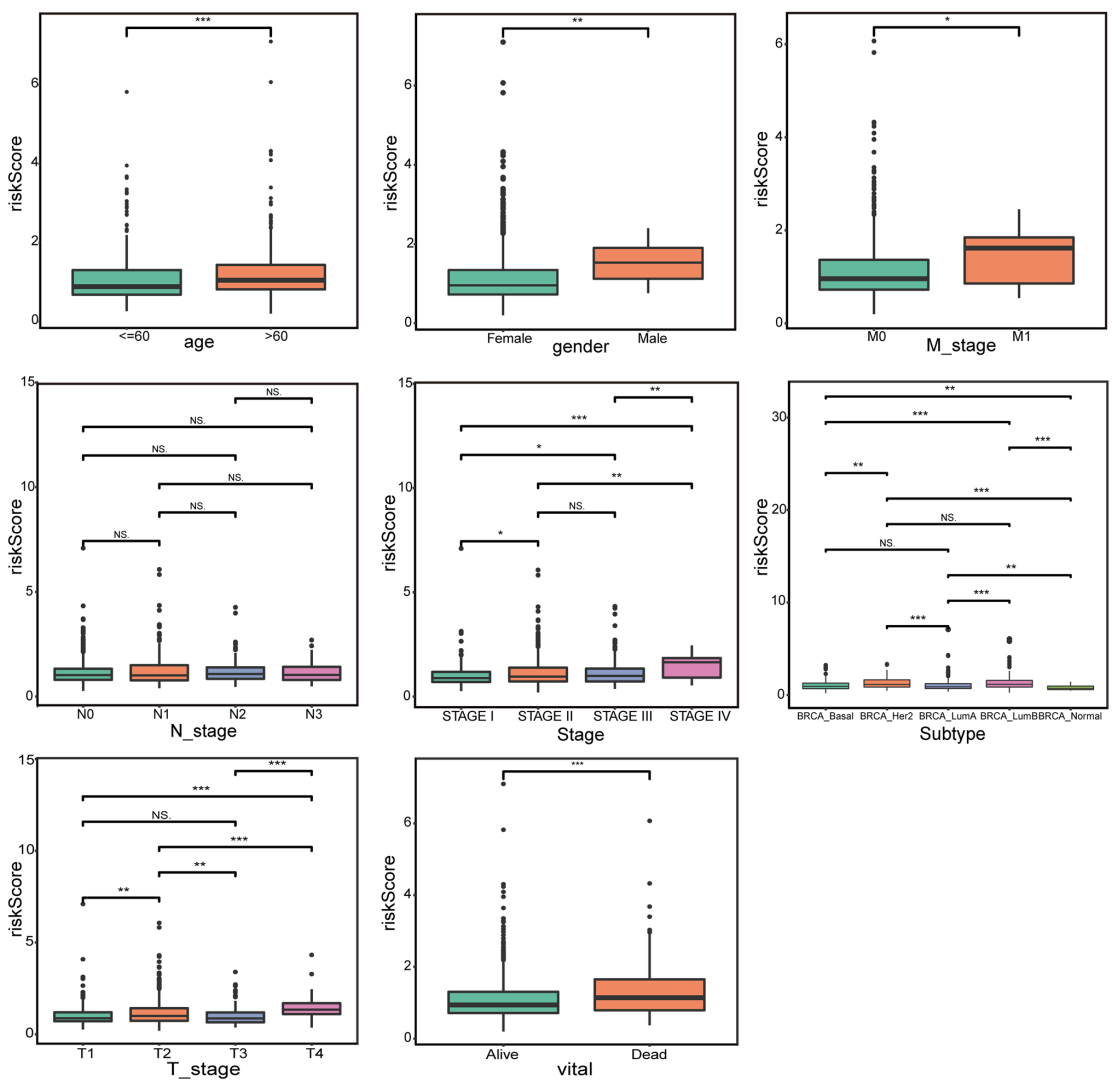
RiskScore difference between subgroups of eight clinicopathological characteristics

Differences of risk score among BRCA individuals with different clinical characteristics were compared for the correlation of the risk model and clinicopathological characteristics using Chi-square test and rank-sum test. The Chi-square test results suggested that except for N (*p* = 0.62), there were significant differences in age, T, M, sex, subtype, vital, and stage between the two risk groups (Table 4). Furthermore, the rank-sum test results demonstrated that the RiskScores differences among the subgroups of T, M, Stage, Vital, Age, Subtype, and Sex groups were significant except for N, indicating the excellent correlations of the risk model and various clinicopathological characteristics (Fig. 6). In addition, the risk stratification analysis was conducted to investigate the application of the risk model in survival prediction in patients with different intrinsic subtypes of BRCA, and it was illustrated that there was a significant difference in OS between different risk groups both Luminal A- and Luminal B-related BRCA cohorts (Supplementary Fig. 2).

**Table 4:** The number of high- and low-risk patients under different clinical factors

	Total	Risk		*p* value
		High	Low	
**Age (year)** Mean (SD)	58.3 (± 13.2)	60.9 (± 13.5)	55.6 (± 12.3)	< 0.001
Gender				
Female	1,057 (98.9%)	524 (98.1%)	533 (99.6%)	0.022
Male	12 (1.1%)	10 (1.9%)	2 (0.4%)	
Vital				
Alive	918 (85.9%)	441 (82.6%)	477 (89.2%)	0.002
Dead	151 (14.1%)	93 (17.4%)	58 (10.8%)	
Stage				
STAGE I	180 (17.1%)	78 (14.9%)	102 (19.4%)	0.05
STAGE II	606 (57.7%)	305 (58.2%)	301 (57.2%)	
STAGE III	245 (23.3%)	127 (24.2%)	118 (22.4%)	
STAGE IV	19 (1.8%)	14 (2.7%)	5 (1.0%)	
M stage				
M0	887 (97.7%)	445 (96.7%)	442 (98.7%)	0.075
M1	21 (2.3%)	15 (3.3%)	6 (1.3%)	
N stage				
N0	502 (47.9%)	250 (47.7%)	252 (48.0%)	0.62
N1	353 (33.7%)	172 (32.8%)	181 (34.5%)	
N2	119 (11.3%)	66 (12.6%)	53 (10.1%)	
N3	75 (7.1%)	36 (6.9%)	39 (7.4%)	
T stage				
T1	276 (25.9%)	116 (21.8%)	160 (29.9%)	< 0.001
T2	620 (58.2%)	328 (61.8%)	292 (54.6%)	
T3	133 (12.5%)	57 (10.7%)	76 (14.2%)	
T4	37 (3.5%)	30 (5.6%)	7 (1.3%)	
Intrinsic subtype				
BRCA Basal	169 (17.5%)	83 (17.2%)	86 (17.7%)	< 0.001
BRCA Her2	75 (7.7%)	50 (10.4%)	25 (5.1%)	
BRCA LumA	496 (51.2%)	214 (44.4%)	282 (58.0%)	
BRCA LumB	193 (19.9%)	126 (26.1%)	67 (13.8%)	
BRCA normal	35 (3.6%)	9 (1.9%)	26 (5.3%)

### Excellent potential of nomogram for clinical prediction

**Fig. 7:**
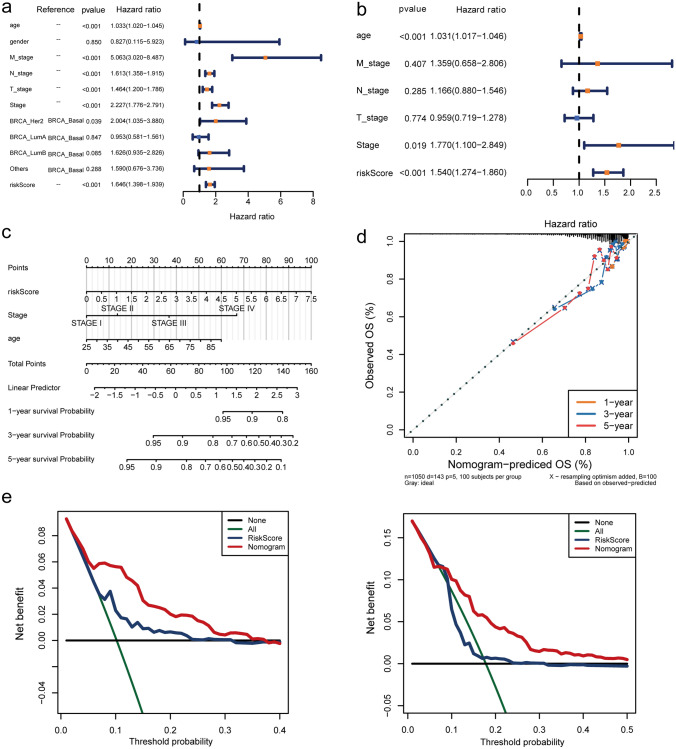
Risk model nomogram construction and verification. Forest plot for clinicopathological characteristics in univariate (**a**) and multivariate (**b**) Cox independent prognostic analyses. **c** The nomogram consists of RiskScore, Stage, and age. **d** Calibration curves for the BRCA patients 1-, 3-, and 5-year OS predictions of the nomogram. **e** Decision curve analysis (DCA) curves comparing the nomogram and RiskScore for the 3-year survival rate of BRCA patients (left) and the 5-year survival rate of BRCA patients (right)

Univariate and multivariate Cox analyses were further used to evaluate the independent prognostic value among the risk model and various clinicopathological characteristics. T, N, M, stage, age, and RiskScore could be considered as independent prognostic factors in the univariate Cox independent prognostic analysis (*p* < 0.05) (Fig. 7a). Next, the multivariate Cox independent prognostic analysis showed that the *p* values of age, stage, and RiskScore were less than 0.05, which could be regarded as independent prognostic factors for BRCA (Fig. 7b). Moreover, a nomogram was constructed for clinical utilization. The C-index of the nomogram based on age, stage, and RiskScore was 0.778, and the slopes of the calibration curves for the nomogram at 1, 3, and 5 years were close to 1, revealing that the prediction of this nomogram was accurate (Fig. 7c, d). At the same time, DCA curves also suggested that the net benefit of the nomogram was greater than that of the risk model (Fig. 7e).

### Correlation between the risk model and the tumor microenvironment

**Fig. 8:**
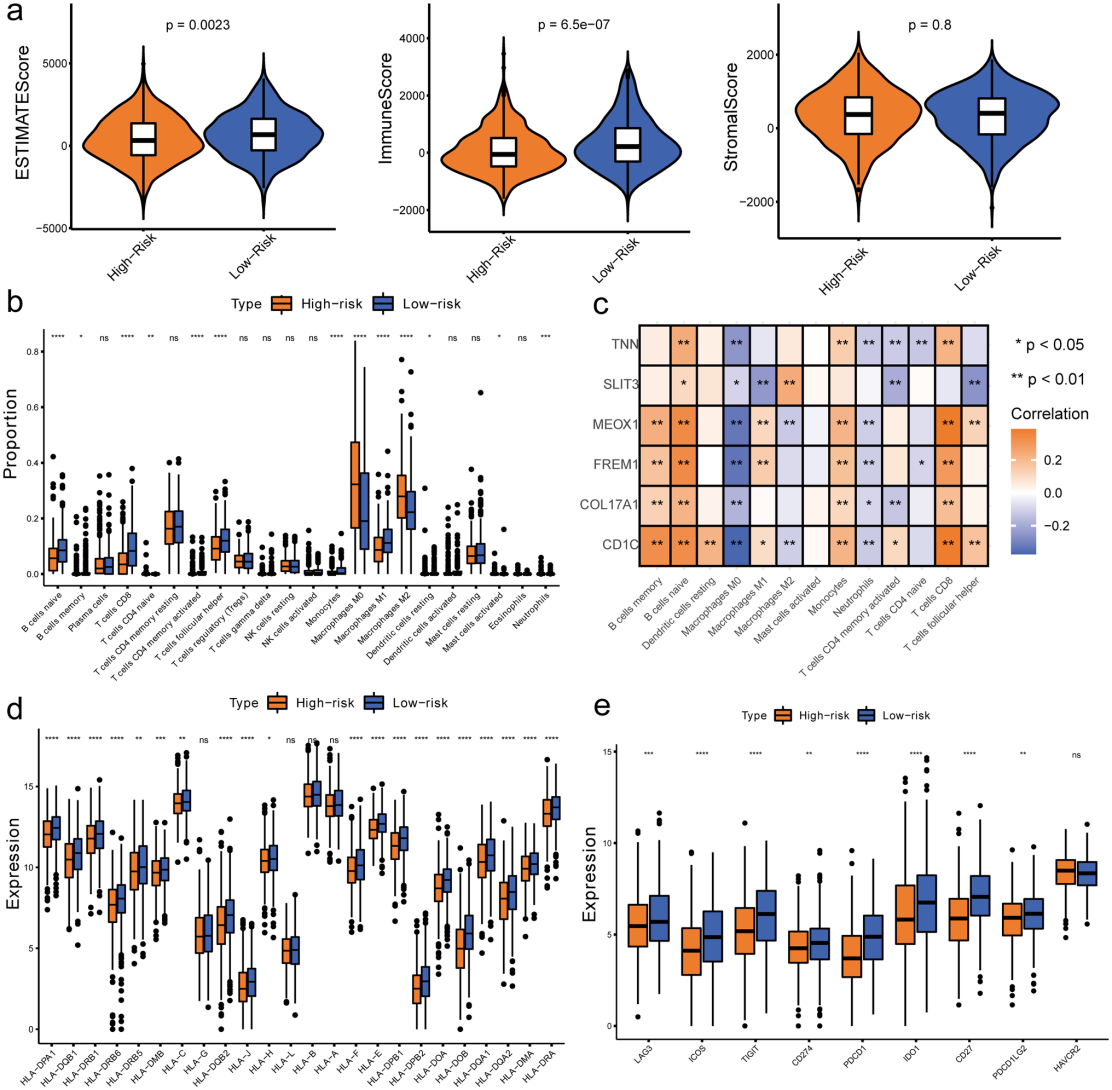
Impact of risk models on immune heterogeneity. **a** Violin plots of differences in the immune microenvironment between the high- and low-risk groups. **b** Boxplot of immune cell infiltration in the two risk groups (*represents *p* < 0.05, **represents *p* < 0.001, ***represents *p* < 0.001, and ****represents *p* < 0.0001). **c** Correlation map between model genes and differential immune cells. **d** Boxplot of HLA-related gene expression differences between the high- and low-risk groups. **e** Boxplot of 9 immune checkpoint molecule expression differences between the high- and low-risk groups

Through a series of approaches of ESTIMATE and CIBERSORT, the relationship between the risk model and the tumor microenvironment was also investigated. The ESTIMATE results revealed that immune scores and ESTIMATE scores between the high- and low-risk groups were notably different (*p* < 0.05) (Fig. 8a). Furthermore, CIBERSORT was applied to calculate the proportions of 22 types of immune cells in 561 BRCA samples (HIGH = 273, LOW = 288) after eliminating samples with *p* > 0.05. The boxplot of the rank-sum test indicated that 13 types of immune cells (CD8 T cells, naive B cells, follicular helper T cells, M0, M1, M2 macrophages, activated memory CD4 T cells, monocytes, neutrophils, naive CD4 T cells, activated mast cells, resting dendritic cells, and memory B cells) were different between the 2 risk groups (Fig. 8b). Moreover, the risk model genes were positively correlated with B cells but negatively correlated with M0 macrophages (Fig. 8c).

Besides, differences of 24 HLA genes as well as 9 immune checkpoint molecules in different risk groups were explored as well through rank-sum test. Results illustrated that 20 out of 24 HLA-related genes (HLA − DPA1, HLA − DQB1, HLA − DRB1, HLA − DRB6, HLA − DRB5, HLA − DMB, HLA − C, HLA − DQB2, HLA − J, HLA − H, HLA − F, HLA − E, HLA − DPB1, HLA − DPB2, HLA − DOA, HLA − DOB, HLA − DQA1, HLA − DQA2, HLA − DMA, and HLA − DRA) were differentially expressed between the two risk groups (*p* < 0.05) (Fig. 8d). Finally, the rank-sum test of nine immune checkpoint molecules revealed that the expression differences between the two risk groups of 8 immune checkpoint molecules (TIGIT, IDO1, LAG3, ICOS, PDCD1LG2, PDCD1, CD27, and CD274) were significant, indicating the therapeutic potential for BRCA by targeting the mechanisms controlling immune checkpoint expression (Fig. 8e).

### SNV of risk model genes and relative expression levels of related genes in normal and primary tumors

**Fig. 9:**
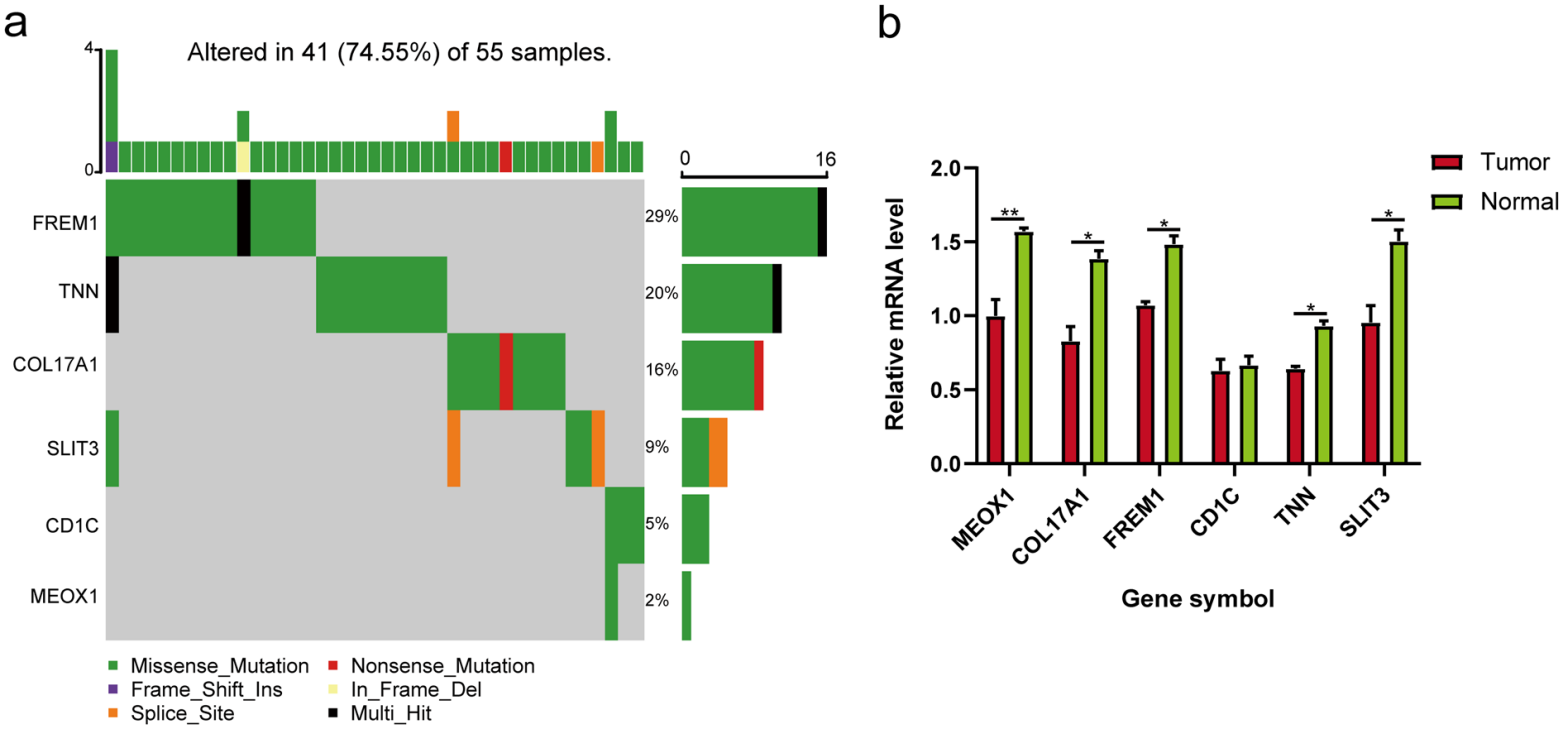
**a** Waterfall plot of mutation SNV types and mutation frequencies of 6 model genes. **b** Validation of model gene expression between breast cancer tissues and normal tissues by qPCR

Finally, the mutation rate of SNVs based on the “maftools” and the mRNA expression levels of 6 model genes in clinical BRCA samples were investigated. The waterfall diagram suggested that a total of 41 samples were altered in at least one model gene, which accounted for 74.55%, and FREM1 was the most mutated model gene with a frequency of 29% followed by TNN (20%) and COL17A1 (16%) (Fig. 9a). To further directly validate the expressions of these model genes in BRCA tissues, we assessed the expression levels of these genes in BRCA tissues and normal tissues by qRT‒PCR, and the results showed that except for CD1C, the other five genes had lower mRNA levels in tumor tissues than in normal tissues (Fig. 9b).

## Discussion

In recent years, RNA modification has become a research hotspot. M^1^A is an important posttranscriptional modification of RNA [14, 45]. Many studies have proven that m^1^A is closely related to the tumor immune response [18, 46]. There are currently approximately 2570 m^1^A modification sites validated in humans [47], but little is known about the relationship between m^1^A modification and BRCA.

In recent years, bioinformatics methods have been widely used in breast cancer research. For example, the expression, somatic mutation, copy-number variation, and biological function of pyroptosis-related genes in breast cancer were evaluated based on the TCGA-BRCA dataset [48]. Yang et al. [35] used the WGCNA method and ssGSEA Z-score to predict survival and risk stratification in triple-negative breast cancer. A prognostic signature based on 6 fatty acid metabolism-related genes relevant to OS was generated using Lasso Cox hazards regression analysis in the TCGA dataset and was validated in two external cohorts [49]. In this study, a prognostic model was constructed by 6 m^1^A-related genes (MEOX1, COL17A1, FREM1, CD1C, TNN, and SLIT3) screened by univariate Cox analysis and LASSO regression analysis.

Considering that breast cancer is intrinsically heterogeneous and different intrinsic subtypes are associated with distinct biological features and clinical outcomes. By comparing the risk scores for different intrinsic subtypes of breast cancer, it was found that the risk scores were well differentiated among different subtypes. Some studies have found that Luminal A and Luminal B may have differences in clinical diagnosis [50], and the risk scores of the two also had significant differences, which preliminarily proves the effectiveness of the study. Moreover, risk stratification survival analysis for each subtype was carried out as well. The results showed that the prognostic model had good predictive ability in the prognosis of Luminal A and Luminal B patients with BRCA. Meanwhile, correlation analysis between risk models and clinical factors was conducted. Except for the N-stage, other clinicopathological features had a significant correlation with risk scores. Although, the significant correlation between risk genes MEOX1, COL17A1, and lymph-node metastasis in triple-negative breast cancer patients has been reported [51, 52]. However, these studies did not involve the specific mechanism of m1A regulation, which is not inconsistent with our conclusions. Although univariate analysis has shown that lymph-node (N) status was associated with OS, further multivariate analysis has shown that it was not sufficient to participate as an independent prognostic factor in the construction of m6A-related nomograms, which was also similar to our findings [53]. We speculated that the relevant mechanism of m1A had less influence on the lymph-node metastasis process of breast cancer patients.

Studies have shown that high levels of MEOX1 were an independent prognostic factor for non-small cell lung cancer (NSCLC), and it could regulate cell proliferation and colony formation in vitro, making it a potential therapeutic target for NSCLC [54]. Yodsurang V mentioned that COL17A1 was a novel p53 transcriptional target in BRCA that inhibited cell migration and invasion and was positively associated with prognosis, and the expression level of COL17A1 mRNA in both primary and metastatic BRCA tissues was significantly reduced, which was consistent with our findings [55]. FREM1 was mainly involved in cellular metabolism and immune cell infiltration. The results of immunohistochemical (IHC) and immunofluorescence (IF) showed that the expression of FREM1 in BRCA tissue was significantly reduced as shown in the current study, and low FREM1 expression was an independent prognostic factor for BRCA [56]. The study by Zhang et al. [57] demonstrated that SLIT3 was a potential tumor suppressor in lung adenocarcinoma. And meanwhile, the results of IHC in a study of BRCA showed that SLIT3 expression was lower than that in normal tissues [58], exhibiting the same expression patterns in this study. Therefore, we believe that these genes may be involved in the prognosis of breast cancer. In this work, the C-index of the m^1^A-related nomogram was calculated to be 0.7782541, indicating that the prediction of the nomogram model was accurate. We performed a decision curve analysis on the risk model and nomogram, and compared the prediction efficiency of the simple risk model and the model after adding clinical factors (M stage). The results showed that the latter can better predict the survival of patients.

It is noteworthy that the DEGs between the high-m^1^A and low-m^1^A risk groups and the low-m^1^A risk groups which may be attributed to the fact that the role of m^1^A modification in tumors is heterogeneous. The repressive impact of m^1^A on translation is probably due to its scarcity in cytosolic mRNAs [17, 59]. For the m^1^A eraser genes ALKBH1 and ALKBH3, it was suggested that the mRNA expression of MFAP2 and the methylation modification of m^1^A were increased in colorectal cancer when ALKBH1 was silenced [60]. The depletion of ALKBH3 enhances the decay of Aurora A mRNA and inhibits its translation [61]. Similarly, the expression of key genes relevant to the ErbB and mTOR pathways could also be inhibited by ALKBH3 in gastrointestinal cancer [62]. On the other hand, overexpression of TRM6/TRM61 mRNA as m^1^A writer genes was detected in highly invasive glioblastoma multiforme, which reduced PKCα [59]. Moreover, it was found that the expression of DEGs in hepatocellular carcinoma patients with different m^1^A methylation modification modes was mostly regulated, which was similar to our results. It was indicated that the inhibition of m^1^A methylation on gene expression may play an important role in BRCA.

A large number of studies have also shown that immune infiltration is associated with tumor prognosis. Tumors with a large amount of CD8+ T-cell infiltration, M1 macrophages, and plasma cells in the tumor microenvironment seem to have a better prognosis [63–67]. Higher levels of M0 and M2 macrophages and lower levels of CD8+ T cells in the tumor microenvironment are associated with poorer prognosis [68, 69]. In our study, risk model genes had a strong negative correlation with M0 macrophages and a strong positive correlation with B cells, which has proven the finding of Zhu [69]. A large number of studies have proven that HLA family genes are closely related to immunotherapy. HLA genes are essential for T lymphocyte activation and antigen presentation [70]. Low HLA-E expression is associated with better overall survival in endometrial cancer [71]. This study found that among the 24 HLA family genes, except HLA-G, HLA-L, HLA-B, and HLA-A, the remaining genes were significantly differentially expressed between the high- and low-risk groups.

Immune checkpoint blockade shows better therapeutic response in many tumor treatments [72]. We therefore analyzed the differential expression of immune checkpoints between the high-risk and low-risk groups. Except for HAVCR2, other immune checkpoints were differentially expressed between the high- and low-risk groups. Our findings suggest that BRCA may be particularly sensitive to combination checkpoint blockade therapy. Studies have shown that high LAG3 expression can induce EGFR-TKI and gefitinib resistance, as well as anti-PD-1 therapy resistance [73]. There are also studies, suggesting that ICOS activation may enhance the effects of inhibitory checkpoint blockade [74]. Ostroumov et al. [75] identified TIGIT as a potential target of immune checkpoint combination therapy by transcriptome analysis. In recent years, numerous studies have shown that cancer cells expressing CD274 may have an impact on regulatory T cells in the tumor microenvironment [76]. PDCD1 is closely associated with TMB, MSI, and immune cell infiltration, and can be used as a prognostic marker in various cancers [77]. A new finding suggested that IDO1 promotes GC metastasis and may be a promising target for GC anticancer therapy [78]. Hence, these immune checkpoints may offer new directions for breast cancer treatment.

Finally, we examined the mRNA expression levels of m^1^A-related genes in breast cancer tissues and normal breast tissues. Except for CD1C, other genes had lower mRNA levels in tumor tissues than in normal tissues. Studies have discovered that over-expression of MEOX2 promoted apoptosis through inhibiting the PI3K/Akt pathway in laryngeal cancer cells [79]. A study by Feiyu Mao et al. demonstrated that high expression of COL17A1 is a marker for predicting poor prognosis in pancreatic cancer and promoting tumor progression through the NF-κB pathway [80]. Another study demonstrated that elevated FREM1 expression in breast cancer is a marker of favorable prognosis and high levels of immune infiltration [56].

However, this study also has limitations. We only verified the mRNA expression of the genes and RIP-qPCR and western blot experiments of six m^1^A methylation-related key genes, especially the CD1C gene, should be completed in a follow-up study. In addition, the exploration of the original cohort data was not involved in this study and required further investigation [58]. Clinical experiments and in vivo and in vitro experiments are required to validate the correlation between m^1^A modification and BRCA, which could make our findings more sound and solid.

In conclusion, an m^1^A RNA methylation regulator-related prognostic model containing six genes (MEOX1, COL17A1, FREM1, CD1C, TNN, and SLIT3) was constructed, and the clinical correlation analysis and construction of the nomogram based on the prognostic model were conducted to provide a theoretical reference for individual counseling and clinical preventive intervention in BRCA. In addition, the prognostic gene expression had significant correlations with M0 macrophages and naive B cells. Various immune checkpoint molecules (ICOS, TIGIT, etc.) that were differentially expressed in the two risk groups might be considered potential therapeutic targets in BRCA. Moreover, mutations in FREM1 and TNN as well as the m^1^A RNA methylation regulator YTHDF1 occurred in the BRCA process. In the future, we will continue to carry out experimental verification.

## Supplementary Information

Below is the link to the electronic supplementary material.

Supplementary file1 (PDF 1916 KB)

## Data Availability

The data that support the findings of this study are available on request from the corresponding author.
