# Supplementary material for: Establishment of a N1-methyladenosine-related risk signature for breast carcinoma by bioinformatics analysis and experimental validation
Source: Breast Cancer. 2023 May 13;30(4):666–84. doi: 10.1007/s12282-023-01458-1 (PMC10284980; doi:10.1007/s12282-023-01458-1)

Breast Cancer

Establishment of a N1-methyladenosine-related risk signature for breast carcinoma by bioinformatics analysis and experimental validation

Leilei Li*[1], Wenhui Yang*[2], Daqi Jia*[1], Shiqi Zheng[1], Yuzhe Gao[3#], Guanghui Wang[3#]

[#]**Corresponding author:**

Guanghui Wang, Department of Breast Surgery, Guizhou Provincial People's hospital, Guizhou 550002, People's Republic of China, Email: wgh 0625@163.com

Yuzhe Gao, Department of Breast Surgery, Guizhou Provincial People's hospital, Guizhou 550002, People's Republic of China, Email: gyzhe2004@126.com

Supplementary Information

Supplementary Fig.1 The Kaplan-Meier (K-M) survival analysis for six model genes.

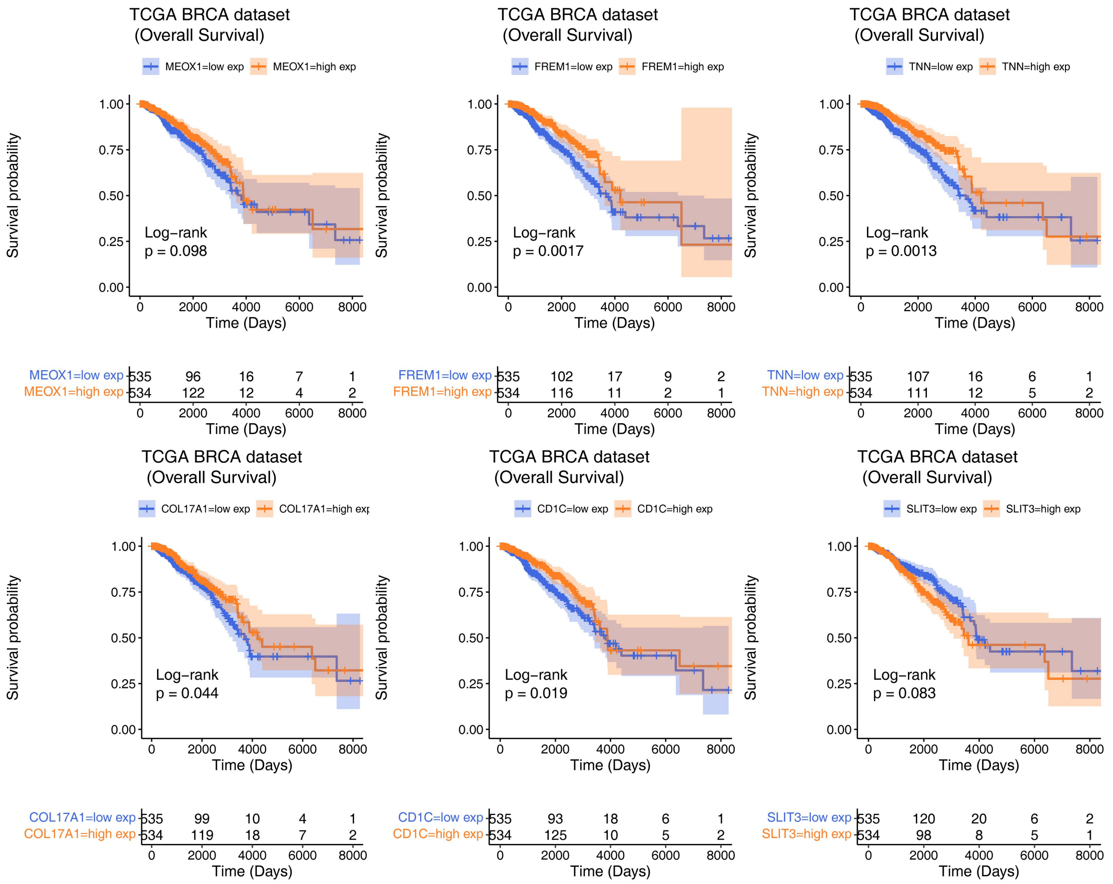

Supplementary Fig.2 The risk stratification analysis in TCGA-BRCA cohorts with different intrinsic subtypes of BRCA.

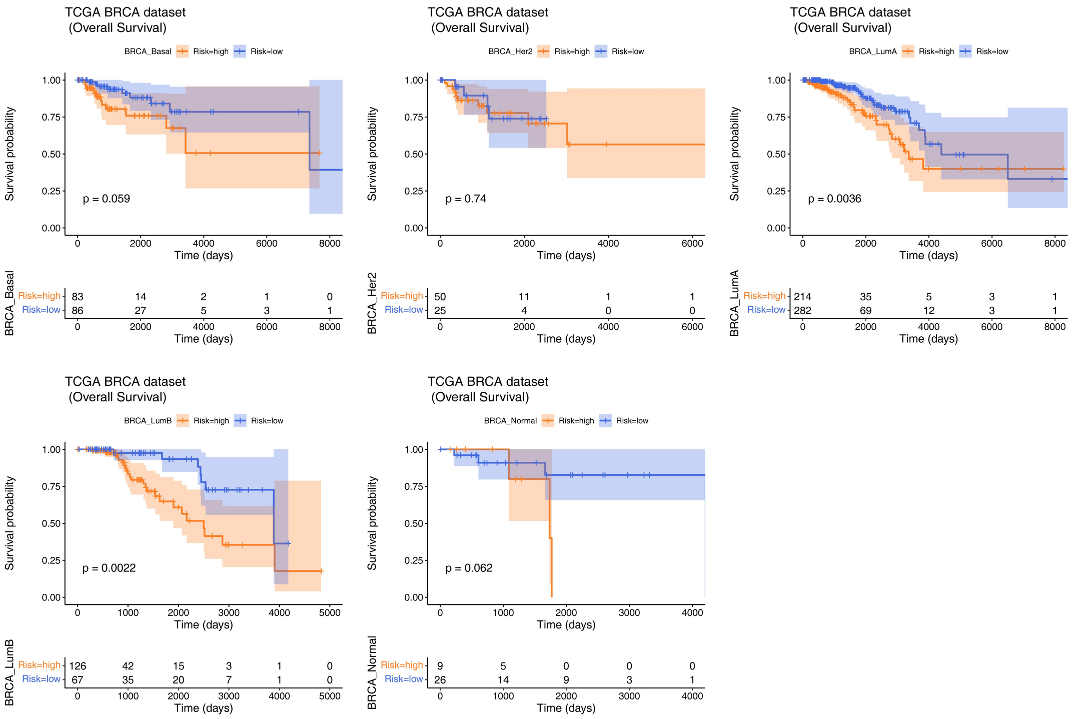

Supplement: Supplementary file 1 — Supplementary file1 (PDF 1916 KB) [file 12282_2023_1458_MOESM1_ESM.pdf]
